# OP79 Innovations In Measuring And Valuing Outcomes: Generating Preferences For The EQ Health And Wellbeing Short

**DOI:** 10.1017/S0266462324001363

**Published:** 2025-01-07

**Authors:** Clara Mukuria

## Abstract

**Introduction:**

The value of health technologies may not be fully reflected in existing generic preference-based measures (GPBMs). The nine-dimension EQ Health and Wellbeing Short (EQ-HWB-S) is a new GPBM developed to assess health, informal carer, and social-care-related quality of life. The objective was to compare standard and new approaches used to generate preferences for the EQ-HWB-S.

**Methods:**

Three feasibility studies that included qualitative work have valued the EQ-HWB-S with members of the public in the United Kingdom: (i) videoconferencing interviews using time trade-off (TTO) and discrete choice experiments (DCEs) from the EuroQol Valuation Technology (EQ-VT) protocol (n=600); (ii) online DCEs using the Potentially All Pairwise RanKings of all possible Alternatives (PAPRIKA) method (n=300), an adaptive DCE with a binary search to locate “dead”; and (iii) Online elicitation of Personal Utility Functions (OPUF) (n=300), which is a compositional method with dimension weighting, response level rating and anchoring on dead. Participant/interviewer feedback, data quality, and the weights were compared.

**Results:**

Self/interviewer reported understanding was high (>70%) across all studies. Qualitative findings indicated misunderstanding for some OPUF steps (e.g., the anchoring on dead). Inconsistencies or illogical answers were small in the EQ-VT study (7%) and OPUF (16%). The PAPRIKA study had a priori exclusions criteria (e.g., time taken) that resulted in 44 percent exclusions. Pain, activity, mobility, and sadness/depression were the most important in all the studies. The value of the worst state was −0.384, −0.51, and −0.15 for EQ-PVT, PAPRIKA, and OPUF, respectively, and there were differences in dimension weights (e.g., PAPRIKA gave less weight to mobility but more to cognition).

**Conclusions:**

EQ-HWB-S is a GPBM that provides an innovative approach to measuring and valuing outcomes. Standard and new approaches to eliciting preferences are feasible, but there are differences in the resultant weights. PAPRIKA and OPUF may improve attribute attendance and be more cost effective as they are administered online, but there is scope for improvement to ensure understanding and engagement.

